# Serum Eicosanoids Metabolomics Profile in a Mouse Model of Renal Cell Carcinoma: Predicting the Antitumor Efficacy of Anlotinib

**DOI:** 10.3389/fimmu.2022.824607

**Published:** 2022-02-09

**Authors:** Ping Du, Lingling Xuan, Ting Hu, Zhuoling An, Lihong Liu

**Affiliations:** Department of Pharmacy/Phase I Clinical Trial & Research Unit, Beijing Chao-Yang Hospital, Capital Medical University, Beijing, China

**Keywords:** eicosanoids, anlotinib, metabolomics, pharmacodynamics, renal cell carcinoma

## Abstract

Anlotinib (ANL) shows promising efficacy in patients with renal cell cancer (RCC). Here, for the first time, a serum eicosanoid metabolomics profile and pharmacodynamics in Renca syngeneic mice treated with ANL was performed and integrated using our previous HPLC-MS/MS method and multivariate statistical analysis. The tumor growth inhibition rates of ANL were 39% and 52% at low (3 mg/kg) and high (6 mg/kg) dose levels, without obvious toxicity. A total of 15 disturbed metabolites were observed between the normal group and the model group, and the intrinsic metabolic phenotype alterations had occurred due to the treatment of ANL. A total of eight potential metabolites from the refined partial least squares (PLS) model were considered as potential predictive biomarkers for the efficacy of ANL, and the DHA held the most outstanding sensitivity and specificity with an area under the receiver operating characteristic curve of 0.88. Collectively, the results of this exploratory study not only provide a powerful reference for understanding eicosanoid metabolic reprogramming of ANL but also offer an innovative perspective for the development of therapeutic targets and strategies, the discovery of predictive biomarkers, and the determination of effective tumor monitoring approaches.

## Introduction

According to data from the 2020 Global Cancer Observatory (GLOBOCAN), renal cell cancer accounts for an estimated 431,288 new diagnoses and 179,368 deaths each year, representing 2.2% of total cancer diagnoses, and 1.8% of all cancer deaths (1). The 5-year survival rate ranges between 12-92.5% according to located stages (2), which means that a large percentage of kidney/renal cancer (RCC) patients still face disease progression. Cancer metabolism is one of the hallmarks and targeting metabolism may be an effective approach for cancer therapy (3–5). Due to mutated, inactivated, or hyper-activated genes in renal tumorigenesis and regulated metabolic reprogramming (e.g., glycolysis, TCA cycle), RCC has been designated as a “Metabolic Disease” (6–8). Over the past decades, eicosanoids and cascade metabolites can regulate various biological processes (e.g., cell proliferation, migration and angiogenesis, invasion, metastasis, inflammatory, and immunology) and play a pivotal role in cancer progression and targeted therapy (9–13).

Anlotinib (ANL) is a novel targeted vascular endothelial growth factor receptor (VEGFR), fibroblast growth factor receptor (FGFR), platelet-derived growth factor receptors (PDGFR), and c-kit, and shows promising efficacy in patients with renal cell cancer (RCC) (14–16). Recently, Pan et al. identified differential serum metabolomics in lung cancer-bearing nude mice treated with ANL or saline (17). Also, our previous studies not only revealed the longitudinal eicosanoids metabolomics of ANL in healthy rats but also elucidated the longitudinal pharmacometabolnomics of ANL for phenotype, efficacy, and toxicity in patients with advanced solid tumors (13, 18). Pharmacometabolomics integrates information of metabolites and drug treatment to facilitate the development of precision medicine, biomarker discovery, and prediction of drug efficacy and toxicity (18, 19). However, to date, there is no eicosanoid metabolomics or metabolic reprogramming studies of ANL for RCC *in vivo* or *in vitro*.

In this study, with the help of our previous reliable HPLC-MS/MS method (13), a serum eicosanoids metabolomics profile was investigated in Renca syngeneic mice, and integration analysis was performed for the antitumor efficacy of ANL. We initially revealed potential biomarkers for predicting the efficacy and toxicity of ANL. The results of this study will enlighten eicosanoid metabolic reprogramming after ANL treatment and offered meaningful references for the discovery of predictive biomarkers and potential therapeutic targets and strategies in RCC.

## Materials and Methods

### Material and Chemicals

Eicosanoids and stable isotope-labeled internal standards (IS) were purchased from Cayman Chemical (Ann Arbor, MI, USA) (**Supplementary Table S1**). Detailed information was shown in our previous literature (13). Anlotinib (purity≧99%) was provided by DC Chemicals (Shanghai, China) and serial diluted with 5% methylcellulose. RPMI-1640 medium, Dulbecco’s modified eagle medium (DMEM), fetal bovine serum (FBS), 0.25% trypsin-EDTA, and penicillin-streptomycin were provided by Thermo Scientific (USA). HPLC grade organic solutions (e.g., acetonitrile, isopropyl alcohol, methanol) were obtained from Fisher Scientific (Pittsburgh, PA, USA). Modifier of mobile phase-formic acid was obtained from DIKMA Co., Inc. (Fairfield, OH, USA). Ultrapure Millipore water was prepared by a purification system.

### Cell Culture

The Renca cell line (mouse kidney, ATCC, Manassas, VA, USA) was cultured in RPMI-1640 medium with 10% FBS 100 units/mL penicillin, 100 μg/mL streptomycin, and 2 mM glutamine at 37°C with 5% CO_2_ and appropriate humidity. The media were changed once every 3-4 days depending on cell confluence. When adherent cells approached convergence, they were digested with trypsin and subcultured. All subsequent experiments were fulfilled using cells in the logarithmic growth phase.

### Animal

Mus Musculus, female BALB/c (5-6 weeks old, 18-22 g) mice were obtained from Beijing Biotechnology Co., Ltd. (Beijing, China). All animals were maintained under SPF conditions (temperature: 23 ± 3°C; humidity: 40-70%, 12 h light/dark cycle) and were housed 5 per cage with *ad libitum* receiving food and water. The animal study was reviewed and approved by the Animal Care and Ethics Committee of Beijing Chao-Yang Hospital, Capital Medical University. All protocols were approved in accordance with guidelines issued by the Guide for the Care and Use of Laboratory Animals.

### Renca Syngeneic Mouse Model

A total of 15 BALB/c mice were used for this Renca syngeneic model. Renca cells were harvested and re-suspended in PBS to 3.0×10^7^ cells/mL. An aliquot of 100 μL tumor cell suspension was injected carefully subcutaneously into the right flank of the back. The mice were randomized by the tumor volume into different treatment groups. After the tumor reached approximately 100 mm^3^, the mice were randomized into three groups (5 mice/group) and administer a gavage (p.o.) of vehicle (saline), ANL treatment (3 mg/kg or 6 mg/kg). The doses were determined based on a previous study (16, 20).

Tumor volume (TV) was obtained as (length × width^2^)/2 by electric caliper measurements. Changes in body weight were monitored twice weekly. The percentage of ΔT/C (% of control for Δgrowth) was calculated using the following formula: (ΔT/ΔC) ×100%, where ΔT and ΔC are the changes in tumor volume (Δgrowth) for the treated and control groups, respectively (21). On the last day of the experiment, all mice were euthanized under carbon dioxide anesthesia, and the tumor tissue was stripped after blood collection.

### HPLC-MS/MS Method of Eicosanoid Metabolomics

A high-performance liquid chromatography-tandem mass spectrometry system (HPLC-MS/MS, Shimadzu, Japan; QTRAP 5500, SCIEX, Canada) was used for eicosanoid metabolomic analysis. The chromatography columns (Waters BEH, C_18_, 2.1×100 mm, 1.7 μm) and elution solvent (gradient elution, 17 min) were all evaluated and employed according to our previous study (13). The ESI source MS parameters were set and employed for quantitation (**Supplementary Table S1**).

A simple protein precipitation method was performed for sample preparation. Briefly, an aliquot of 20 μL serum was added with 10 μL ISs and 50 μL MeOH for precipitation. The supernatants were infused into HPLC-MS/MS system after centrifugation. This bioanalytical method was fully validated with the calibration linear range of 0.005-500 ng/mL for 68 eicosanoids (13).

### Integration Analysis of Metabolomics and Efficacy of Anlotinib

**Figure 1:**
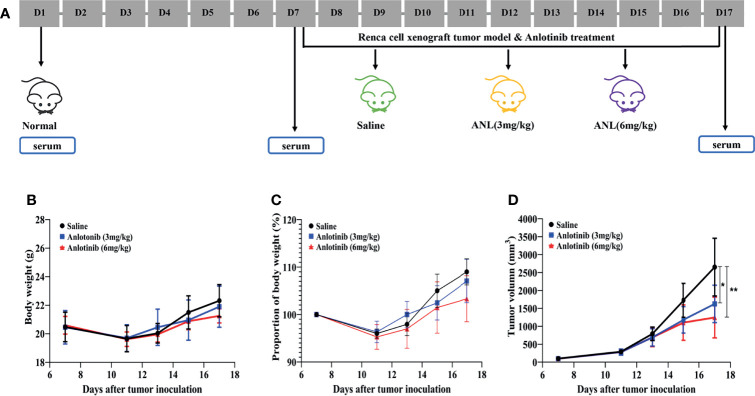
*In vivo* antitumor activities of anlotinib in a Renca cell syngeneic model in mice. **(A)** The timeline of the animal experiment. **(B)** Bodyweight. **(C)** Bodyweight variance. **(D)** Relative tumor volume at indicated time points (*n*=5). Data are shown as means ± SEM. *p < 0.05, **p < 0.01 *vs* saline.

Serum samples were collected according to the following experimental stage: healthy mice (normal), model stage (Renca syngeneic), and ANL treatment (3 mg/kg or 6 mg/kg). Eicosanoid metabolomics was evaluated using previous serum samples (**Figure 1A**). Besides, the antitumor efficacy and toxicity of ANL (e.g., tumor volume, body weight) were also assessed and compared on the final day of the experiment.

### Data Processing and Statistical Analysis

For the raw metabolomic or antitumor efficacy data processing, MultiQuant 3.0.1 software was applied. SIMCA-P software (v14.1, Umetric, Umeå, Sweden), MetaboAnaylst 5.0 (http://www.metaboanalyst.ca) and IBM SPSS 26.0 (Armonk, New York, United States) were used to build mathematic models or analysis, such as unsupervised principal component analysis (PCA), supervised orthogonal projection to latent structures squares-discriminant analysis (OPLS-DA), partial least squares (PLS), hierarchical cluster analysis (HCA), Student’s t-test and Pearson correlation. The compounds with values of variable importance in the projections (VIP) >1 or statistical significance of p<0.05 or correlation coefficient of 0.5 were picked out for further identification and metabolic analysis. To check overfitting and random effects, 200 permutation tests were used to assess the predictive ability. The p-value <0.05 was considered statistically significant.

## Results

### Antitumor Efficacy of Anlotinib in a Renca Syngeneic Model

**Figure 2:**
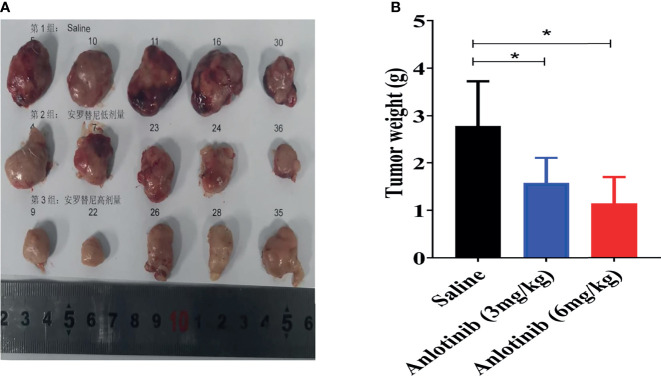
The typical tumors harvested from mice sacrificed on the last day of experiment. **(A)** The photographs of tumors on the final day. **(B)** Tumor weight of different groups. *p < 0.05.

As shown in **Figure 1A**, the workflow of this study was designed and implemented over 17 days. After acclimatizing for a few days, approximately 50 µl of serum was collected from healthy stage mice (D1, Normal). Afterward, a Renca syngeneic model was developed using logarithmic growth phase cells (D7), and the serum was collected before ANL treatment (Model) and on the final day of the experiment (ANL). Regarding the body weight and changes, following 10 consecutive days of ANL drug treatment, no statistically significant differences were observed among all groups (**Figures 1B** **, C**), indicating no apparent toxicity was found. The TVs of all groups were also displayed in order to elucidate the antitumor efficacy of ANL. As described in **Figure 1D** and **Table 1**, the TVs of ANL treatment groups (3 mg/kg or 6 mg/kg) were significantly smaller than that in the saline group (p<0.05, p<0.001), and the tumor growth inhibition rates (TGI) were 39% and 52% for ANL low and high dosage, respectively. Furthermore, the tumor tissues of all groups were harvested and weighed. Compared with the saline group, the tumor tissue weight was lighter in the ANL-treated group (**Figure 2**). Collectively, these data supported that ANL potentiated an antitumor effect in Renca syngeneic model in mice.

**Table 1:** Overview of tumor growth inhibition *in vivo* in Renca syngeneic model in mice.

Treatment	Tumor volume (mm^3^)		T/S (%)	TGI (%)	P value^a^
	Pre-drug (D7)	Post-drug (D17)			
Saline	99 ± 3	2658 ± 361	–	–	—
ANL (3 mg/kg)	99 ± 5	1630 ± 234	61%	39%	0.018
ANL (6 mg/kg)	98 ± 4	1250 ± 255	48%	52%	0.002

T/S: ratio of treatment/saline; Tumor cell growth inhibition rate (TGI): 1 -T/C; a compared with saline group.

### Eicosanoids Metabolic Profiles Among Different Treatment

**Figure 3:**
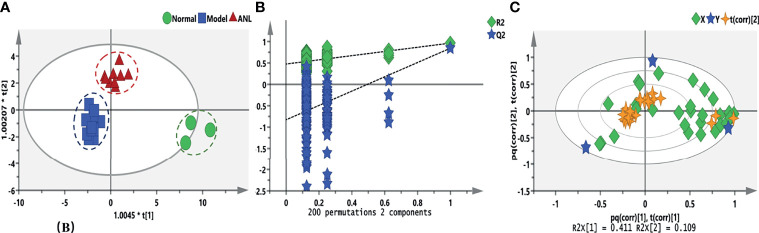
The eicosanoids metabolic profiles among different treatments. **(A)** OPLS-DA score plots of three groups. **(B)** Random permutation test with 200 iterations. **(C)** The correlation bioplot of OPLS-DA model.

After evaluating the efficacy of ANL, eicosanoid metabolomics was investigated under different treatments. **Figure 3** shows the metabolic trajectory and correlation analysis. a total of 31 analytes were determined and compared. From the results of **Figure 3A**, serum samples of different treatments were divided into close clusters, which demonstrated that the metabolic fingerprint of the same group was comparatively stable. Moreover, this OPLS-DA model was verified by 200 permutation tests, and no overfitting was observed (**Figure 3B**, R^2^= (0.0, 0.475), Q^2^= (0.0, -0.829)). The biplot (**Figure 3C**) overlaid scores and loading scatter plots together for correlation analysis between variables. The metabolites correlated positively or negatively, respectively. In Renca syngeneic model, a total of 15 disturbed metabolites were observed between the normal group and model group when set VIP>1, p<0.05 (**Supplementary Table S2**).

### Pharmametabolomics of Anlotinib

**Figure 4:**
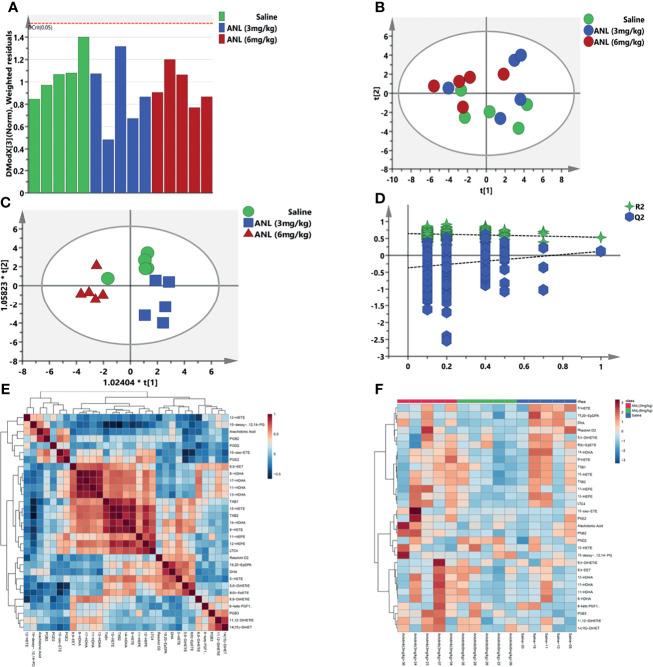
The eicosanoid metabolomic profiles of anlotinib. **(A)** DModX curve. **(B)** PCA. **(C)** OPLS-DA. **(D)** Permutation test with 200 iterations. **(E)** Differential metabolite correlation heatmaps. **(F)** HCA of eicosanoid metabolomic profiling.

Based on previous results, the eicosanoid metabonomic fingerprint of ANL was employed and analyzed at low and high dosages. For data quality analysis, a Distance to model (DModX) curve was used to inspect the outliers. As shown in **Figure 4A**, all serum samples were within the range of 2. **Figures 4B, C** illustrated all metabolic data from the PCA and OPLS-DA models. The individuals from the three groups were well distinguished without distinct overfitting (**Figure 4D**, R^2^ = (0.0, 0.643; Q^2^= (0.0, -0.366), permutation test with 200 iterations). The positive or negative correlations among metabolites were shown in (**Figure 4E**). Heatmap clustering analysis (HCA) was also conducted to examine metabolic disturbance caused by ANL treatment (**Figure 4F**).

**Figure 5:**
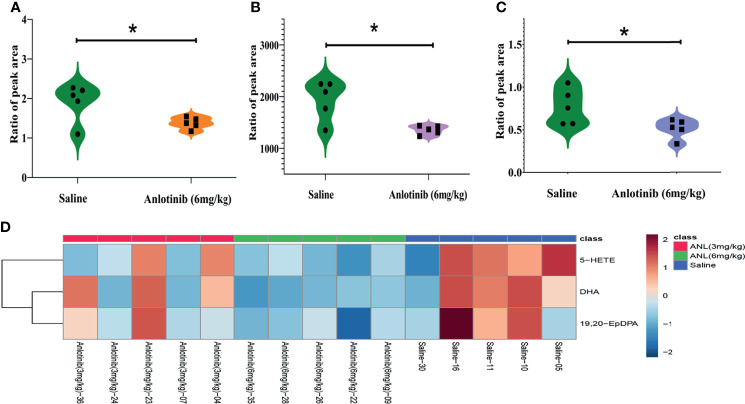
The three significant changed metabolites in Renca cell syngeneic mice treated by anlotinib (6 mg/kg) when the cutoffs were set as VIP>1 and p<0.05. **(A)** 5-HE-TE. **(B)** DHA. **(C)** 19,20-EpDPA. **(D)** The heatmap of the above-mentioned metabolites. *p < 0.05.

As described in **Figure 5**, although the ANL exerted an antitumor effect at a low dose level (3 mg/kg), no significant changes in metabolites were observed compared to the saline-treated group. However, the ANL high-dose group (6 mg/kg) caused a significant reduction in three metabolites (5-HETE, DHA, 19,20-EpDPA). Overall, these metabolic results echoed the previous pharmacodynamic results to some degree, and the intrinsic metabolic phenotype changes had occurred as a result of ANL.

### Correlation Analysis Between Metabolomics and Efficacy of Anlotinib

**Figure 6:**
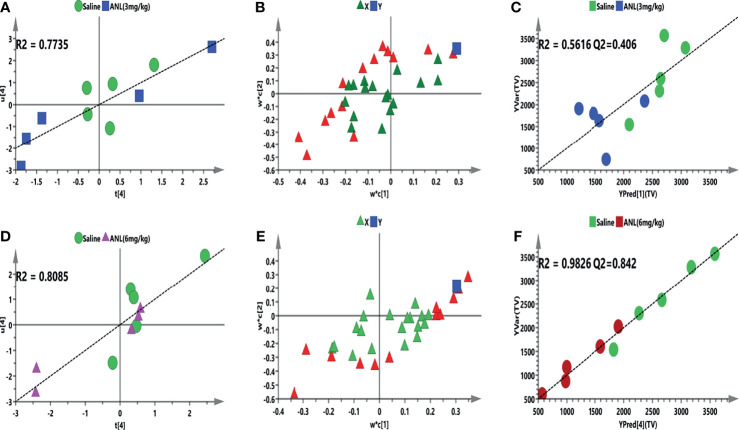
The supervised PLS model for correlating metabolic profiles with the efficacy of anlotinib. **(A, D)** are initial PLS for the first latent variables (ratio of peak area of metabolites, *X* block) of tumor volume (ANL, 3 mg/kg and 6 mg/kg, *Y* block) prediction model, respectively. **(B, E)** are loading plots for tumor volume, respectively. The square (blue) means the response variable; each triangle represents a metabolite, and the triangles (red) show the metabolites with VIP > 1.0. **(C, F)** are refined models to predict tumor volume based on the screened biomarkers.

To further elucidate whether eicosanoid metabolic profiling accompanies the antitumor effect of ANL, Pearson correlation analysis and supervised PLS model were constructed to identify which metabolites highly interact with this trend. Firstly, an unsupervised PCA model was employed to find out outliers. All mice are distributed according to different metabolic profiles (**Supplementary Figure S1**). Secondly, the peak area ratios of 31 metabolites were correlated to TV in the initial PLS model to approximately study the relationship between the *X* (ratio of peak area of metabolites) and *Y* (TV) variables. A four-component PLS model was constructed for TV, which demonstrated a positive correlation (**Figure 6A**, R^2^ = 0.7735; **Figure 6D**, R^2^ = 0.8085). **Figures 6B. E** show the loading plot and the correlation between the predictive variable (*X*, triangle) and the response variable (*Y*, box) at low and high dosages of ANL. Besides, 14 (ANL-3 mg/kg) and 12 (ANL-6 mg/kg) VIP>1.0 *X* variables were recognized because of the contribution of *X* variables to the PLS model (red triangles), which were chosen for the following prediction of efficacy. Afterward, the Pearson correlation analysis was adopted for the association between TV and VIP>1.0 variables. Regarding the forecasting efficiency, 2 or 6 VIP>1.0 screened variables were significantly interrelated with TV and used for refined PLS analysis, respectively (**Table 2**). As illustrated in **Figure 6C**, a refined PLS model was built based on previous variables, which help explain about 56.2% variation (R^2^Y) and predict 40.6% variation (Q^2^) for TV of ANL-3 mg/kg treatment. In the meantime, as shown in **Figure 6F**, it could explain about 98.3% variation (R^2^Y) and predict 84.2% variation (Q2) for TV of ANL-6 mg/kg treatment based on these 6 variables. Collectively, the results demonstrated that this PLS model displayed the ability to predict the efficacy of ANL with no risk of overfitting. Variables listed in **Table 2** were considered a potential biomarker for predicting antitumor efficacy.

**Table 2:** Identified metabolites of high VIP values in initial PLS Models correlating to tumor volume.

Model type	Compounds	VIP	Correlation coefficient
Tumor volume-ANL (3 mg/kg)	PGB3	1.56893	-0.546
	PGE2	1.52857	-0.601
Tumor volume-ANL (6 mg/kg)	19,20-EpDPA	1.39511	0.657
	PGB3	1.35468	-0.612
	5,6-DiHETrE	1.3272	0.615
	14-HDHA	1.15085	0.505
	PGD2	1.91087	-0.711
	DHA	1.57149	0.735

### Prediction of Tumor Volume Based on Significant Metabolites

**Figure 7:**
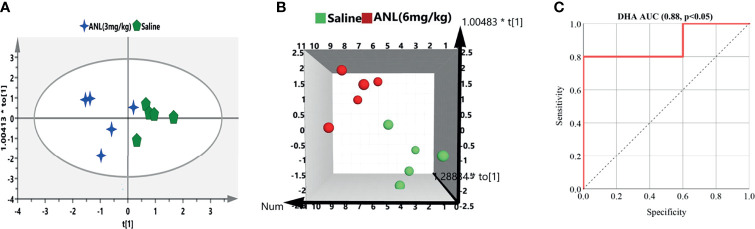
The OPLS-DA to differentiate the efficacy groups based on the screened biomarkers. Green shape marks indicate mice treated by saline **(A)** or **(B)**, blue stars mean mice treated by ANL [3 mg/kg, **(A)**], and red circle represents mice treated by ANL [6 mg/kg, **(B)**]. **(C)** The ROC curve of DHA for the ANL (6 mg/kg) group.

For the purpose of verifying the predictive capability of the potential biomarkers selected above, different treatment groups were separated into two groups (control, drug treatment) in the light of their TV. Discrimination between saline and ANL treatment groups based on the screened biomarkers was performed using OPLS-DA models. As illustrated in **Figure 7**, the picked 2 biomarkers (ANL-3 mg/kg *vs* saline) and 6 biomarkers (ANL-6 mg/kg *vs* saline) completely distinguish the two groups, which indicate that these potential biomarkers can discriminate antitumor efficacy of ANL. Furthermore, the AUC value of the receiver operating characteristic (ROC) curve was 0.88 for DHA. The sensitivity and specificity were 0.8 and 0.6, respectively. These results suggest that DHA could be as a potentially useful metabolite for predicting the efficacy of ANL (6 mg/kg) in Renca syngeneic mice.

### Quality Control Processes

In the light of obtaining reliable results, multiple strategies, and sources of variations (e.g., sample preparation, HPLC-MS/MS status) have been evaluated and taken to minimize undesirable deviations. As displayed in **Supplementary Figure S2**, the peak area ratios of pooled QC samples were gathered after PCA, which demonstrated that the fluctuations in the mixed QC samples associated with each analyte are small and constant.

## Discussion

The main purpose of this study was to investigate the interplay between the eicosanoid metabolic profiling and antitumor efficacy in Renca syngeneic mice. As far as we know, no research is currently accessible regarding the eicosanoid metabolic agitation and the interrelation between the metabolic fingerprint and pharmacodynamics of ANL.

For the first time, our work has shown that ANL significantly inhibited Renca syngeneic tumor growth without adverse effects (**Figure 1**). As shown in **Figures 1B, C**, ANL did not cause significant changes in body weight in the syngeneic mice model. Compared with the control saline group, both low and high dosages of ANL significantly inhibited the increase of tumor volumes (**Figure 1D**). It is widely reported that ANL plays a critical role in several types of cancer with favorable safety, such as refractory metastatic soft-tissue sarcoma (22), locally advanced or metastatic medullary thyroid cancer (23), colorectal cancer (24). A multicenter, randomized phase II trial reported by Zhou et al. (14) assessed the clinical efficacy of ANL and sunitinib as first-line treatment for patients with metastatic renal cell carcinoma (mRCC). The clinical efficacy (e.g., progression-free survival, overall survival, objective response rate, and disease control rate) of ANL was similar to that of sunitinib, but with a more favorable safety profile and fewer adverse events (AEs) of grade 3 or 4. Recently, a single-arm, open-label, phase 2 study investigated the efficacy and toxicity of ANL as second-line treatment for patients with mRCC after prior one VEGFR-TKI. The results demonstrated a promising clinical efficacy with mild adverse effects (15). Although the TGI of ANL treatment was 39% and 52% (**Table 1**, and **Figure 2**), the combination of clinical trials and the results herein confirmed that ANL is safe and effective in the treatment of RCC.

Metabolic interactions with microenvironments and metabolic reprogramming are the key hallmarks for tumorigenesis (3). Due to the alteration of the von Hippel-Lindau (*VHL*) gene and activation of the Ras-PI3K-AKT-mTOR pathway, the RCC is occasionally considered as a “Metabolic Disease”; besides, the reprogramming of the fatty acid metabolism is one of the most associated pathways (8). It is well known that eicosanoids, biologically active lipids, have been involved in tumor evolution, progression, and metastasis (9). The lipid mediators metabolized from three major metabolic pathways of eicosanoids (the cyclooxygenase (COX), the lipoxygenase (LOX), and the cytochrome P450) play a critical role in immunoregulation, tumor immune microenvironment, crosstalk between metabolic reprogramming, and efficacy (9, 25, 26). In this study, the metabolic trajectories of the model group were different from those of the normal group when modeled by no-overfitting OPLS-DA (**Figure 3A**). Emerging evidence suggests that metabolites of LOX (e.g., HETEs, LTs), COX (e.g., PGE2, PGD2, TXs), and CYP450 (e.g., EETs, DHETs) are involved in carcinogenesis (27, 28), the results of this study indicated that the metabolites of TXB1, TXB2, PGB2, and others were significantly disturbed (**Supplementary Table S2**). The eicosanoids, especially those generated by CYP enzymes (e.g., EETs) and soluble epoxide hydrolase (e.g., DHETs and DHDPs), were related to the endothelial cell proliferation and angiogenesis processes (12). Moreover, ANL could inhibit angiogenesis by suppressing the activation of VEGFR2, PDGFRβ, and FGFR1. The dynamic trend of metabolite disturbances shows that ANL can correct the disturbance of eicosanoid metabolites caused by tumor growth to a certain extent (**Figure 3**).

Regarding the eicosanoids metabolomic fingerprints treated by ANL in Renca syngeneic mice, metabolites for each group almost assembled with a high quality of metabolomics data (**Figure 4A**). On the last day of the experiment, the serum samples were collected and analyzed for the metabolomic study. Although the metabolic changes of the low dose group (ANL, 3 mg/kg) were not found, some metabolites, such as 5-HETE, DHA, and 19,20-EpDPA, were significantly decreased compared with those of the saline group (**Figure 5**). The tumor microenvironment and metabolism of RCC are receiving increasing attention (8, 29–32). By analyzing 46 snap-frozen primary renal cell carcinomas and their corresponding normal renal cortex biopsies, the 5-LOX protein levels and metabolites of 5-HETE were found to be significantly elevated in RCC (33). The DHA and its metabolite of 19,20-EpDPA were significantly decreased after ANL (6 mg/kg) treatment. Possible reasons for the decrease include a limited sample size. Overall, the eicosanoids metabolic fingerprint was disturbed and changed due to ANL administration, and the results may be used for further understanding the mechanism of ANL.

After evaluating the antitumor efficacy of ANL, a correlation analysis was firstly performed and elucidated between the efficacy and the eicosanoid metabolomics. A total of 31 metabolites were included and correlated with TV to check whether a regression was found. After checking the outliers, initial and refined PLS models were constructed between the metabolites and TV. The metabolic characteristics were extremely correlated with TV after ANL treatment, and several significant disturbance metabolites were observed and further utilized for predicting the efficacy of ANL (**Figure 6**). A positive linear regression (**Figure 6A**, R^2^ = 0.7735; **Figure 6D**, R^2^ = 0.8085) was shown. **Figures 6B, E** indicates the loading plot, and the relationship between the predictive variable (*X*, triangle) and the response variable (*Y*, box). The *X* variables on the top right or low left corner suggested positively or negatively correlated to TV, respectively. As illustrated in **Figures 6C, F**, a PLS model was built in view of the previous eight variables, which help explain the 56.2-98.3% variation (R2Y) and predict the 40.6-84.2% variation (Q^2^) for TV. Permutation tests were performed with 200 iterations in order to avoid overfitting of the prediction model (data not shown). In order to examine this potential biomarker, an ROC curve was performed and analyzed. The AUC of DHA was 0.88 with p<0.05, which indicates that DHA may be a potential predictive biomarker. Overall, the results indicated that the predictive model exerted a capability to estimate TV. Also, the expression of the enzymes 5-LOX and 15-LOX2 is strengthened in RCC cells compared to normal renal cells (33), and LOX can promote the secretion of immunosuppressive chemokine CXCL2 and cytokine IL 10 to regulate immune-evasion of RCC cells (34). Variables listed in **Table 2** were considered as potential biomarkers for predicting TV. Although limited samples were used in this study, several metabolite biomarkers were firstly discovered and provided to predict the efficacy of ANL.

As far as we know, no available study is published regarding the eicosanoids metabolic profiles after ANL treatment in Renca syngeneic mice model. As a novel multi-targeting tyrosine kinase inhibitor of ANL, little is known about the correlation between metabolic profiles and efficacy. On the one hand, in Renca syngeneic mice, we have shown the antitumor effect of ANL and elucidated the integrative analysis between pharmacodynamics and pharmacometabolomics. On the other hand, the results of this study not only provide a powerful reference for understanding metabolic reprogramming of eicosanoids but also offer an innovative perspective for the development of therapeutics, the discovery of predictive biomarkers, and the recognition of effective tumor monitoring approaches.

Several limitations should be mentioned. Firstly, the sample size of this exploratory research was small and more data may be needed to pool and verify these results. Thus, external validation cohort should be supplemented to better illustrate the relationship between efficacy and metabolomics. Secondly, due to the availability of blood samples from RCC patients, we only investigated the antitumor efficacy and metabolomics profile in Renca syngeneic mice. Thirdly, in this study, only eicosanoid metabolomics in the serum of different groups of mice were examined, and metabolomic features in the local tumor microenvironment (stripped tumors) were not examined. However, in view of the many advantages of non-invasive, convenient, real-time, and high-throughput biomarkers for predicting the efficacy of ANL in the treatment of RCC, this study did not use the stripped tumor as the main sample for detection, but rather predicted the antitumor efficacy of ANL by the determination of eicosanoid metabolites in blood. Taken together, to enlighten the mechanism of ANL in RCC, our work firstly revealed the antitumor efficacy and eicosanoid metabolic trajectories induced by ANL, and explored the predictive drug efficacy biomarkers in Renca syngeneic mice, which will provide a notable scientific contribution to prevention or therapy in patients with RCC.

## Conclusion

Taken together, for the first time, we have uncovered the interplay between antitumor efficacy of ANL and comprehensive eicosanoid metabolic alterations in Renca syngeneic mice and revealed the potential predictive biomarker for efficacy or toxicity. This study reveals the eicosanoid metabolic profiles of mice in the healthy, disease model, and drug treatment states. Besides, a correlation analysis between eicosanoid metabolomics and efficacy of ANL was firstly explored, and eight metabolites (especially for DHA) were considered as potential serum biomarkers to predict the TV of different treatments. Overall, the results of this study not only provide a powerful reference for understanding metabolic reprogramming of eicosanoids but also offer an innovative perspective for the development of therapeutics, the discovery of predictive biomarkers, and the detection of effective tumor monitoring approaches.

## Data Availability Statement

The original contributions presented in the study are included in the article/**Supplementary Material**. Further inquiries can be directed to the corresponding authors.

## Ethics Statement

The animal study was reviewed and approved by the Animal Care and Ethics Committee of Beijing Chao-Yang Hospital, Capital Medical University.

## Author Contributions

Conceptualization, investigation, and writing-original draft: PD. Methodology: TH and LX. Writing-review and editing: TH and ZA. Resources and supervision: LL. All authors contributed to the article and approved the submitted version.

## Conflict of Interest

The authors declare that the research was conducted in the absence of any commercial or financial relationships that could be construed as a potential conflict of interest.

## Publisher’s Note

All claims expressed in this article are solely those of the authors and do not necessarily represent those of their affiliated organizations, or those of the publisher, the editors and the reviewers. Any product that may be evaluated in this article, or claim that may be made by its manufacturer, is not guaranteed or endorsed by the publisher.
